# Utility of the oxygen pulse in the diagnosis of obstructive coronary artery disease in physically fit patients

**DOI:** 10.14814/phy2.15105

**Published:** 2021-11-12

**Authors:** Bradley J. Petek, Timothy W. Churchill, J. Sawalla Guseh, Garrett Loomer, Sarah K. Gustus, Gregory D. Lewis, Rory B. Weiner, Aaron L. Baggish, Meagan M. Wasfy

**Affiliations:** ^1^ Division of Cardiology Massachusetts General Hospital Boston Massachusetts USA; ^2^ Cardiovascular Performance Program Massachusetts General Hospital Boston Massachusetts USA

**Keywords:** cardiopulmonary exercise testing, coronary artery disease, exercise testing, O_2_ pulse, oxygen pulse

## Abstract

Cardiopulmonary exercise testing (CPET) guidelines recommend analysis of the oxygen (O_2_) pulse for a late exercise plateau in evaluation for obstructive coronary artery disease (OCAD). However, whether this O_2_ pulse trajectory is within the range of normal has been debated, and the diagnostic performance of the O_2_ pulse for OCAD in physically fit individuals, in whom $\dot{V}O_{2}$ may be more likely to plateau, has not been evaluated. Using prospectively collected data from a sports cardiology program, patients were identified who were free of other cardiac disease and underwent clinically‐indicated CPET within 90 days of invasive or computed tomography coronary angiography. The diagnostic performance of quantitative O_2_ pulse metrics (late exercise slope, proportional change in slope during late exercise) and qualitative assessment for O_2_ pulse plateau to predict OCAD was assessed. Among 104 patients (age:56 ± 12 years, 30% female, peak $\dot{V}O_{2}$ 119 ± 34% predicted), the diagnostic performance for OCAD (n = 24,23%) was poor for both quantitative and qualitative metrics reflecting an O_2_ pulse plateau (late exercise slope: AUC = 0.55, sensitivity = 68%, specificity = 41%; proportional change in slope: AUC = 0.55, sensitivity = 91%, specificity = 18%; visual plateau/decline: AUC = 0.51, sensitivity = 33%, specificity = 67%). When O_2_ pulse parameters were added to the electrocardiogram, the change in AUC was minimal (−0.01 to +0.02, *p* ≥ 0.05). Those patients without OCAD with a plateau or decline in O_2_ pulse were fitter than those with linear augmentation (peak $\dot{V}O_{2}$ 133 ± 31% vs. 114 ± 36% predicted, *p* < 0.05) and had a longer exercise ramp time (9.5 ± 3.2 vs. 8.0 ± 2.5 min, *p* < 0.05). Overall, a plateau in O_2_ pulse was not a useful predictor of OCAD in a physically fit population, indicating that the O_2_ pulse should be integrated with other CPET parameters and may reflect a physiologic limitation of stroke volume and/or O_2_ extraction during intense exercise.

## Graphical abstract

Cardiopulmonary exercise testing (CPET) guidelines recommend analysis of the oxygen (O_2_) pulse for a late exercise plateau in evaluation for obstructive coronary artery disease (OCAD). The diagnostic performance of the O_2_ pulse for OCAD in physically fit individuals, in whom $\dot{V}O_{2}$ may be more likely to plateau, has not previously been evaluated. We found that a plateau in O_2_ pulse was not a useful predictor of OCAD in a physically fit population and may reflect a physiologic limitation of stroke volume and/or O_2_ extraction during exercise rather than a pathologic fall in stroke volume due to ischemia.

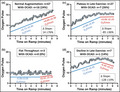

## 1 INTRODUCTION

Cardiopulmonary exercise testing (CPET) offers a broad approach to evaluate exertional symptoms, (Ross et al., 2016) and the addition of ventilatory gas exchange to standard exercise testing incrementally improves diagnostic utility for a variety of cardiovascular and pulmonary conditions (Balady et al., 2010; Chaudhry et al., 2018). The oxygen (O_2_) pulse is a CPET parameter calculated from the ratio of oxygen consumption ($\dot{V}O_{2}$) to heart rate (HR) and, as reflected by the Fick equation, represents the product of stroke volume (SV) and O_2_ extraction. Because O_2_ extraction is typically thought to increase linearly and predictably across the spectrum of exercise capacity, (Stringer et al., 1985; Sullivan et al., 1989) the trajectory of the O_2_ pulse may be interrogated to gain insight into the change in SV during a graded exercise effort (Accalai et al., 2020; Bhambhani et al., 1994; Crisafulli et al., 2007). In theory, the development of exercise‐induced myocardial ischemia may lead to left ventricular (LV) dysfunction, loss of SV augmentation, and a plateau or fall in O_2_ pulse during exercise (Balady et al., 2010).

Driven by the fact that electrocardiogram (ECG)‐only exercise testing suffers from only moderate sensitivity and specificity for the diagnosis of obstructive coronary artery disease (OCAD), (Gianrossi et al., 1989; Morise & Diamond, 1995) the European Association for Cardiovascular Prevention and Rehabilitation (EACPR) and American Heart Association (AHA) Clinical Recommendations for CPET have advocated that O_2_ pulse trajectory evaluation may be a useful addition to ECG testing (Committee & EACPR: Guazzi M, 2012; Mezzani et al., 2009). However, clinical application of O_2_ pulse assessment is limited by a lack of consensus regarding normal SV response to exercise (Vella & Robergs, 2005) as well as debate, on the basis of preliminary results, as to whether the O_2_ pulse is a reliable reflection of SV (Sarma et al., 2014). Others have investigated whether a plateau in the O_2_ pulse curve during exercise improves the diagnostic capability of CPET for OCAD with conflicting results (Belardinelli et al., 2003, 2014; De Lorenzo et al., 2017, 2018; Klainman et al., 1996, 2002; Laukkanen et al., 2006; Munhoz et al., 2007). Also, cohorts in prior studies were not reflective of the population of physically fit individuals that may present for CPET, in whom physiologic plateaus in CPET parameters such as $\dot{V}O_{2}$ (Lucia et al., 2006) and/or O_2_ pulse (Perim et al., 2011) may alter the diagnostic performance of O_2_ pulse‐based metrics for OCAD.

We hypothesized that a plateau in the O_2_ pulse would be less specific for the diagnosis of OCAD in a population with above average fitness and that, in this context, O_2_ pulse parameters would fail to improve upon the diagnostic performance of the exercise ECG in the evaluation for OCAD. To evaluate these hypotheses, we analyzed prospectively‐collected data from a single, high‐volume cardiopulmonary exercise laboratory to identify patients without other relevant forms of cardiac disease who had undergone both clinically‐indicated CPET and coronary angiography. Our primary goal was to determine the incremental diagnostic yield for OCAD of both quantitative and qualitative metrics reflecting O_2_ pulse trajectory.

## 2 MATERIALS AND METHODS

### 2.1 Study population

**FIGURE 1:**
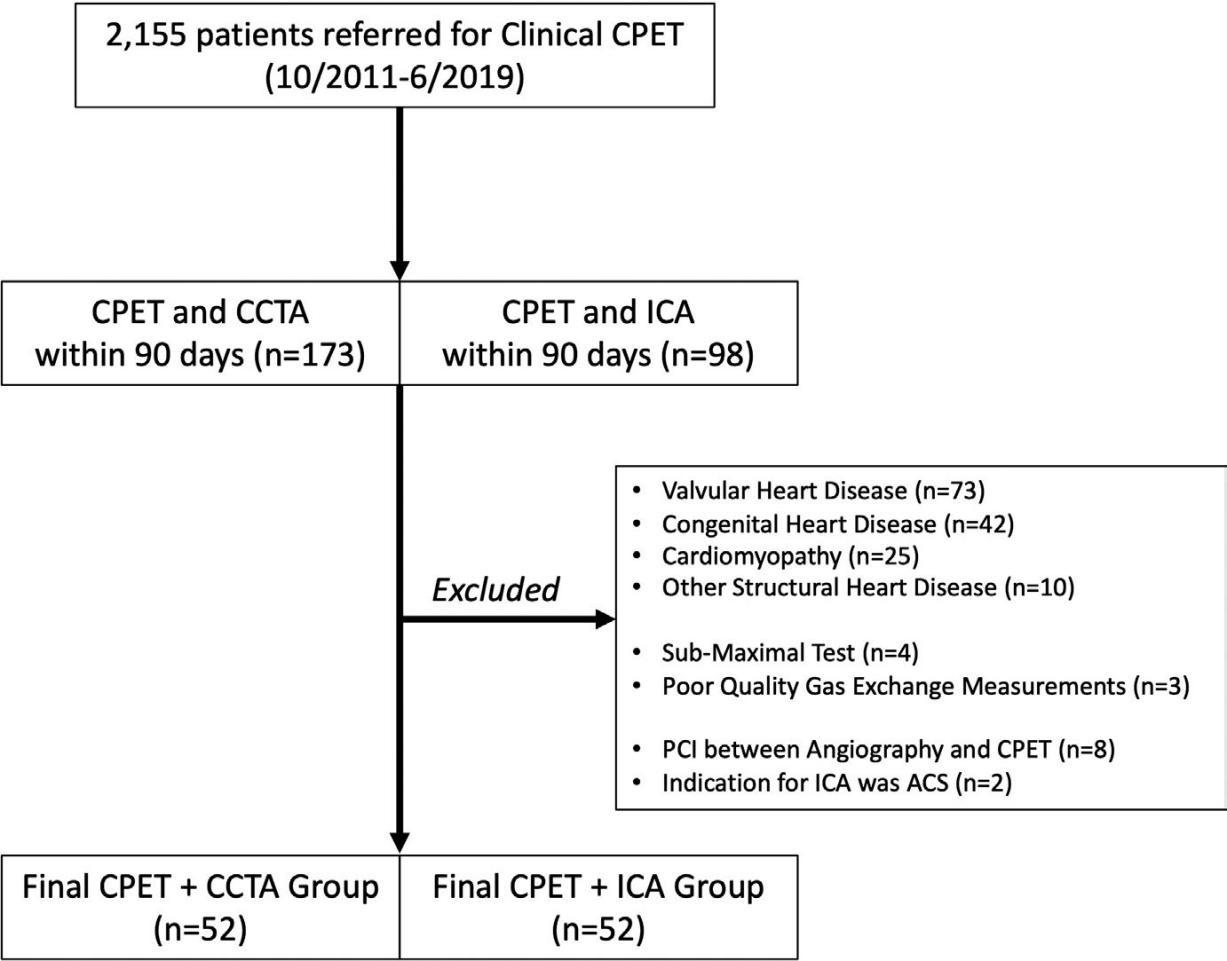
Flow Diagram for Study Inclusion. ACS, acute coronary syndrome; CPET, cardiopulmonary exercise test; CCTA, coronary computed tomography angiography; ICA, invasive coronary angiography; PCI, percutaneous coronary intervention

Participants were eligible for inclusion in this study if they were ≥18 years old and performed CPET on the treadmill or cycle ergometer in the exercise lab of the Massachusetts General Hospital (MGH) Cardiovascular Performance Program (CPP) (Boston, MA, USA) within 90 days of clinically‐indicated invasive coronary angiography (ICA) or coronary cardiac computed tomography (CCTA). Rigorous clinical exclusion criteria (Figure 1) were used to generate a cohort that was free of cardiac disease that might impact SV augmentation, and in whom it could be reasoned that coronary anatomy remained stable between the CPET and angiography. We excluded patients with: (a) history of cardiomyopathy, congenital, or structural heart disease, (b) greater than mild valvular disease, (c) indication for coronary angiography of acute coronary syndrome, (d) coronary revascularization between angiography and CPET if CPET performed after angiography. We also excluded patients with test characteristics that interfered with interpretation of the O_2_ pulse, including (a) significant arrhythmia during CPET, (b) respiratory exchange ratio <1.05, (c) poor quality gas exchange measurements, or (d) short ramp duration (<4 min) of the exercise protocol.

Coronary angiography was performed at the discretion of participants’ clinicians based on clinical history and/or CPET results. The CPP provides clinical care to physically active individuals with suspected or confirmed cardiovascular disease, and patients undergo a standardized maximal effort‐limited CPET on referral unless clinically contraindicated. From exercise laboratory opening (October 1, 2011) through June 1, 2019, CPET results were prospectively collected in a research database. All aspects of this study were approved by the Partner's Human Research Committee (Boston, Massachusetts). Patients or the public were not involved in the design, or conduct, or reporting, or dissemination plans of the research.

### 2.2 Cardiopulmonary exercise test methods

Intensity graded, maximal effort‐limited exercise tests were performed with continuous gas exchange on the treadmill (Woodway Pro 27, Woodway USA, Waukesha, Wisconsin) or upright cycle ergometer (Sport Excalibur Bicycle Ergometer, Lode, Holland). The cycle ergometry protocol consisted of 3 min of free‐wheel cycling followed by continual increase in resistance (10–40 watts/min). Treadmill tests began with a 10‐min warm‐up at 3.0 to 7.5 miles/h and 1% grade followed by a progressive increase in incline (0.5% grade increase every 15 s) at a fixed speed. The intensity of the cycle ergometry ramp and the speed of treadmill testing were determined by the exercise physiologist and participant with a goal of an 8–12 min ramp time. All tests proceeded until exhaustion, onset of limiting symptoms, or development of a clinical contraindication to exercise (Gibbons et al., 2002).

Gas exchange was measured on a breath‐by‐breath basis using a commercially available metabolic cart (Ultima Cardia O_2_; Medgraphics Diagnostics, St. Paul, Minnesota) and analyzed using Breeze Suite software (Medgraphics Diagnostics, Version 8.2, 2015). Peak gas exchange parameters (p$\dot{V}O_{2}$, O_2_ pulse) are reported as the highest average over a period of 30 s during the last minute of effort‐limited exercise. Predicted p$\dot{V}O_{2}$ was calculated by the Jones equations (Jones et al., 1985; Shephard, 1969). For the purposes of generating O_2_ pulse curve slopes, the timing of peak work and p$\dot{V}O_{2}$ were defined by careful test inspection of the breath‐by‐breath data for each participant. The timing of peak work (i.e., peak watts or incline) was corroborated by observation of minute ventilation, $\dot{V}{\text{CO}}_{2}$, and HR. Rarely and limited to treadmill tests, when there was a clear decrease in two or more of these parameters prior to when peak work was recorded, peak work time was moved earlier for purposes of O_2_ pulse analysis. Visual inspection of plotted breath‐by breath $\dot{V}O_{2}$ was used to qualitatively assess when the slope of $\dot{V}O_{2}$ through the end of exercise was approximately zero or less than zero, reflecting $\dot{V}O_{2}$ plateau, and for the purposes of O_2_ pulse analysis, the timing of peak $\dot{V}O_{2}$ was considered the beginning of this $\dot{V}O_{2}$ plateau. A true $\dot{V}O_{2}$ plateau was defined as greater than 30 s. All O_2_ pulse curves and slope fits were inspected, and outlier values in the breath‐by‐breath data (>2 standard deviations from the fitted slope) were excluded and slopes were re‐inspected to assure they accurately reflected the shape of O_2_ pulse curves. Quantitative and qualitative analyses in this study were performed using the breath‐by‐breath data without averaging or smoothing data other than removing outlier values as described above.

Exercise testing was performed with 12‐lead ECG monitoring (Mortara Instrument X12+), and measurement of blood pressures using manual sphygmomanometer (Myers et al., 2009). The ECG response to exercise was considered ischemic as per guidelines (Gibbons et al., 2002).

### 2.3 Quantitative O_2_ pulse trajectory assessment

All participants underwent an intensity graded, maximal effort‐limited exercise test with continuous gas exchange monitoring on the treadmill or upright cycle ergometer. Individual breath‐by‐breath gas exchange data were extracted and plots of the O_2_ pulse curve ($\dot{V}O_{2}$/HR) for each patient were created from the start of CPET ramp until peak work and peak $\dot{V}O_{2}$ (p$\dot{V}O_{2}$). Both peak work and p$\dot{V}O_{2}$ were assessed given p$\dot{V}O_{2}$ may reach a physiologic plateau prior to peak work in a proportion of athletes (Lucia et al., 2006). Slopes of the O_2_ pulse curves were calculated using breath‐by‐breath data for (a) the start of ramp until the final 2 min of exercise (Early Exercise Slope), and (b) the final 2 min of exercise to p$\dot{V}O_{2}$ (Late Exercise Slope). The proportional change in slopes is defined as (Late Exercise Slope – Early Exercise Slope) / Early Exercise Slope. The same assessment was also performed through peak work.

### 2.4 Qualitative O_2_ pulse trajectory assessment

**FIGURE 2:**
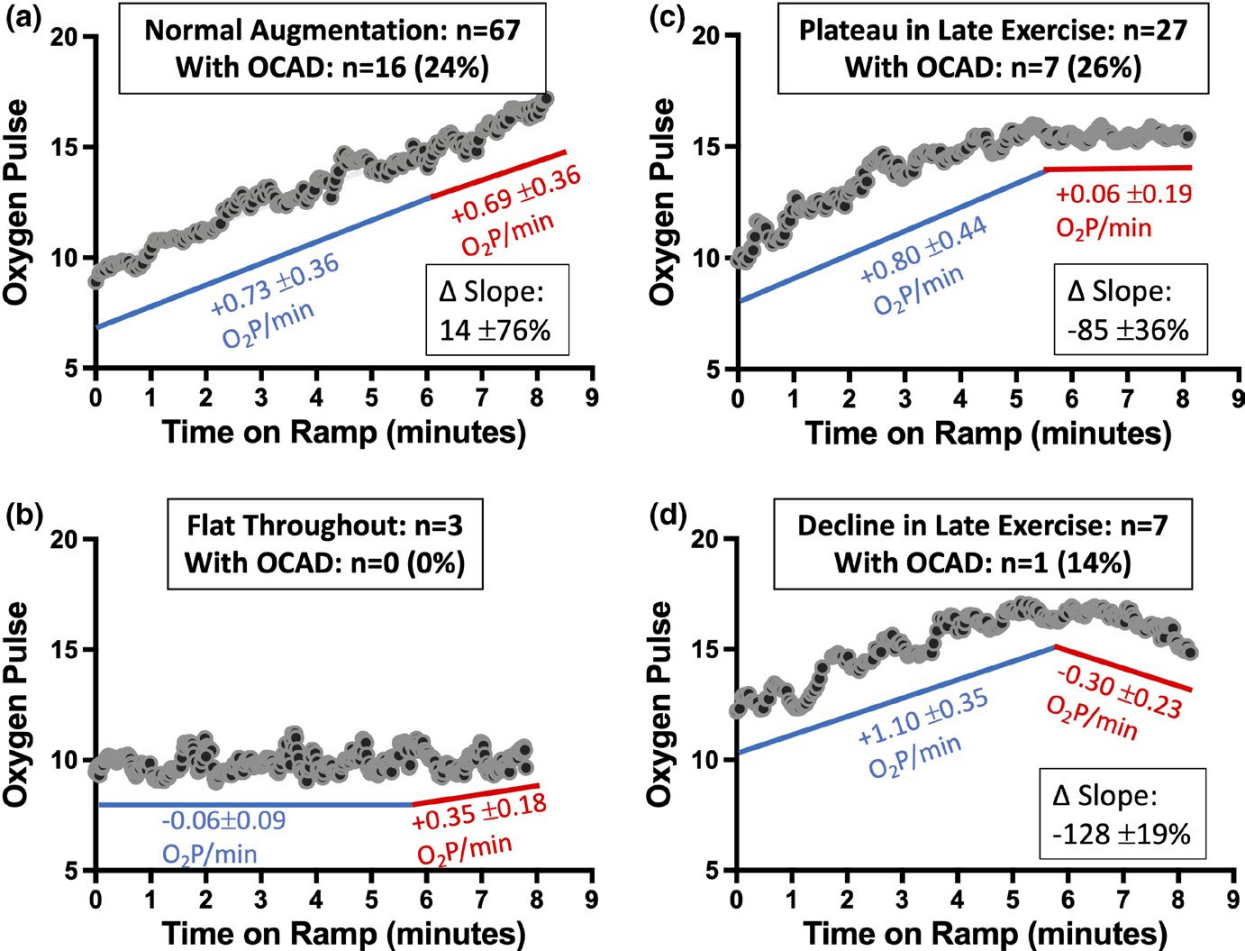
Qualitative Assessment of O_2_ Pulse Trajectory. Over the work ramp portion, CPETs were categorized as demonstrating (a) normal linear O_2_ pulse augmentation through peak $\dot{V}O_{2}$, (b) no augmentation, (c) a plateau in late exercise, or (d) a decline in late exercise. The number of tests overall, the number of tests in patients with OCAD, the average early and late exercise O_2_ pulse slopes, and the proportional change in slope in late versus early exercise are shown for each category

Qualitative assessment of O_2_ pulse trajectory was performed by two physician reviewers (B.P. and T.C.) who were blinded to patient data including angiography. Tests were categorized into four groups (Figure 2): Category A) Normal Augmentation: augmentation of O_2_ pulse during the entire ramp, Category B) Flat Throughout: no augmentation of the O_2_ pulse during the ramp, Category C) Plateau in Late Exercise: initial augmentation of the O_2_ pulse with flattening during late exercise, Category D) Decline in Late Exercise: initial augmentation of the O_2_ pulse with down‐sloping during late exercise. If the two reviewers assigned the same test to different categories, a third physician reviewer (M.W.), blinded to the initial categorization, adjudicated the study.

### 2.5 Definition of CAD

Angiograms were performed as clinically‐indicated at a single center (MGH), and clinical reports were used to assess the presence of CAD. The presence of CAD (“any CAD”) was defined as any luminal irregularities on ICA or CAD‐RADS>0 on CCTA (Cury et al., 2016). OCAD was defined as ICA with ≥70% lesion and/or positive fractional flow reserve (FFR) or instantaneous free‐wave ratio (iFR) by conventional metrics (De Bruyne et al., 2012; Götberg et al., 2017), or as CAD‐RADS‐4 on CCTA (estimated stenosis severity of 70–99%). Multivessel OCAD was defined as OCAD in ≥2 of the 3 epicardial coronary territories.

### 2.6 Statistical analysis

Descriptive continuous variables are presented as mean (SD) or median [IQR] as appropriate and were compared between groups using a two‐sample unpaired *T*‐test or Wilcoxon rank sum test, respectively. Categorical variables are presented as n (%) and compared by the Chi‐square test or Fischer's exact test when n ≤ 5 per category. Univariate predictors for an abnormal O_2_ pulse trajectory were assessed using logistic regression. The diagnostic performance for OCAD of O_2_ pulse metrics alone and in combination with the ECG was assessed via receiver operator characteristic curve analysis. Statistical analysis was performed with R: A Language and Environment for Statistical Computing (R Core Team 2021) and SAS (Version 9.4). O_2_ pulse curve slopes were generated in Graph Pad Prism (version 8.4.1).

## 3 RESULTS

### 3.1 Study population

Among 2,155 patients who were referred for clinically‐indicated CPET, 104 patients met study inclusion criteria (Figure 1). Baseline characteristics are presented in Table 1. The cohort was 56 ± 12 years old with a high proportion of Caucasian (94%) and male (70%) participants. Those completing the CPET on the treadmill had shorter height and lighter weight (*p *< 0.05) but similar body mass index as those on the cycle ergometer. Baseline hemoglobin levels were available in 60% of the cohort and 5 male (Hgb <13) and 1 female athlete (Hgb <12) met criteria for anemia.

**TABLE 1:** Patient characteristics

Characteristic	Cycle Ergometry (n = 65)	Treadmill (n = 39)
Age (years)	57.9 (11.3)	54.1 (14.0)
Female	16 (25)	15 (39)
Race		
Caucasian	63 (97)	35 (90)
Hispanic	0 (0)	2 (5)
Asian	1 (2)	0 (0.0)
Other	1 (2)	2 (5)
Weight (kg)	81.0 (15.4)	74.2 (14.0)^a^
Height (cm)	175.3 (9.9)	170.0 (14.3)^a^
BMI (kg/m^2^)	26.3 (4.1)	26.0 (6.7)
Medical history		
Hypertension	27 (42)	10 (26)
Diabetes Mellitus	5 (8)	0 (0)
Hyperlipidemia	27 (42)	15 (39)
Smoking	17 (26)	10 (26)
Known Ischemic Heart Disease^b^	8 (12)	6 (15)
Prior Stroke	1 (2)	0 (0)
Paroxysmal Atrial fibrillation	8 (12)	4 (10)

Note

Data presented as n (%) or mean (SD).

^a^ *p *< 0.05 for cycle ergometry versus treadmill tests.

^b^ Defined as prior myocardial infarction or percutaneous coronary intervention.

### 3.2 Cardiopulmonary exercise test characteristics

The average p$\dot{V}O_{2}$ was 35.2 ± 12.3 ml/kg/min or 119 ± 34% predicted, and 52% of participants had ischemic ECG changes with exercise. Participants completed tests on the treadmill had higher peak HR (169 ± 18 vs. 154 ± 26 bpm) and peak $\dot{V}O_{2}$ (41.3 ± 10.5 vs. 31.5 ± 11.9 ml/kg/min) than those on the cycle ergometer (*p *< 0.05, Table 2). Average peak O_2_ pulse (Table 2) and other quantitative O_2_ pulse metrics (Table 3) were similar on cycle ergometer and treadmill tests. A total of 31 athletes had mild exercise‐induced arterial hypoxia (EIAH, S_p_O_2_ <95%), 9 athletes had moderate EIAH (S_p_O_2_ <93%), and no athletes had severe EIAH (S_p_O_2_ <88%) (Dempsey & Wagner, 1999).

**TABLE 2:** Cardiopulmonary exercise test characteristics

Characteristic	Cycle ergometry test (n = 65)	Treadmill (n = 39)
Exercise Length to Peak Work (min)	13.1 (2.5)	15.8 (3.0)^a^
Ramp Length to Peak Work (min)	10.1 (2.5)	6.5 (1.9)^a^
Exercise Length to Peak $\dot{V}O_{2}$ (min)	12.9 (2.5)	15.6 (3.0)^a^
Ramp Length to Peak $\dot{V}O_{2}$ (min)	9.9 (2.5)	6.3 (1.9)^a^
Peak RER	1.19 (0.10)	1.15 (0.09)^a^
Peak $\dot{V}O_{2}$ (ml/kg/min)	31.5 (11.9)	41.3 (10.5)^a^
Peak $\dot{V}O_{2}$ Percent Predicted	111 (38)	132 (23)^a^
$\dot{V}O_{2}$/Work (ml/min/W)	8.9 (1.5)	N/A
Peak HR (bpm)	154 (26)	169 (18)^a^
Peak O_2_ Pulse (ml/beat)	16.7 (5.9)	18.0 (4.7)
Peak SBP (mmHg)	189 (34)	180 (33)
Peak DBP (mmHg)	78 (12)	71 (11)^a^
Ischemic ECG Changes with Exercise^b^	32 (53)	19 (50)

Note

Data presented as n (%) or mean (SD).

Abbreviations: DBP, diastolic blood pressure; ECG, electrocardiogram; HR, heart rate; RER, respiratory exchange ratio; SBP, systolic blood pressure.

^a^ *p *< 0.05 for cycle ergometry versus treadmill tests.

^b^ Percentage of those with ischemic ECG changes shown relative to those with interpretable ECGs (Cycle Ergometry Tests: n = 60, Treadmill Tests: n = 38).

**TABLE 3:** Cardiopulmonary exercise test characteristics versus angiography results

Test characteristic	Angiography results			
	No CAD	Any CAD	Obstructive CAD	Multi‐vessel obstructive CAD
	(n = 36, 35%)	(n = 68, 65%)	(n = 24, 23%)	(n = 9, 9%)
Exercise ECG^b^				
Ischemic changes with exercise	14 (40)	37 (59)	18 (82)^a^	5 (71)
Quantitative CPET Characteristics^d^				
Peak RER	1.16 (0.1)	1.18 (0.1)	1.18 (0.1)	1.21 (0.1)
Peak $\dot{V}O_{2}$ (ml/kg/min)	38.0 (13.4)	33.7 (11.5)	34.2 (10.5)	33.0 (5.2)
Peak $\dot{V}O_{2}$, Percent Predicted	120 (37)	118 (33)	118 (30)	109 (14)
$\dot{V}O_{2}$/Work (ml/min/W)^c^	8.9 (2.0)	8.9 (1.3)	9.0 (1.2)	9.1 (1.2)
Peak O_2_P (ml/beat)	17.3 (7.2)	17.1 (4.4)	17.4 (4.2)	17.7 (2.8)
Peak O_2_P, Percent Predicted	125 (37)	121 (27)	121 (25)	114 (14)
O_2_P/time slope over late exercise (ml/beat/min)	0.52 (0.45)	0.40 (0.47)	0.40 (0.45)	0.29 (0.44)
Proportional change in O_2_P/time slope in early vs. late exercise	−0.14 (0.78)	−0.27 (0.85)	−0.38 (0.57)	−0.59 (0.44)
Qualitative Assessment of O_2_P Trajectory^e^				
Category A: Normal augmentation	25 (69)	42 (62)	16 (67)	4 (44)
Category B: Flat throughout	3 (8.3)	0 (0)^a^	0 (0)	0 (0)
Category C: Plateau in late exercise	7 (19)	20 (29)	7 (29)	5 (56)
Category D: Decline in late exercise	1 (3)	6 (9)	1 (4)	0 (0)
Category C or D: Decline or plateau in late exercise	8 (22)	26 (38)	8 (33)	5 (56)

Note

Data presented as n (%) or mean (SD).

Abbreviations: ECG, electrocardiogram; O_2_P, arterial oxygen pulse; RER, respiratory exchange ratio.

^a^ *p *< 0.05 for CAD group compared to no CAD as the reference group.

^b^ Percentage of those with ischemic ECG changes shown relative to those with interpretable ECGs (No CAD: n = 35, Any CAD: n = 63, Obstructive CAD: n = 22, Multi‐Vessel Obstructive CAD: n = 7).

^c^ Peak $\dot{V}O_{2}$/Work only calculated for cycle ergometry tests (No CAD: n = 20, Any CAD: n = 45, Obstructive CAD: n = 17, Multi‐Vessel Obstructive CAD: n = 6).

^d^ Late exercise is defined as the last 2 min of test prior to achievement of peak $\dot{V}O_{2}$. Early exercise is defined as the portion of the test from the beginning of the ramp to the last 2 min. The proportional change in slopes is defined as (Late Exercise Slope – Early Exercise Slope) / Early Exercise Slope. This was not calculated for Category B (Flat Throughout) because slopes approached zero.

^e^ Chi‐squared test or Fisher's exact test (as appropriate) comparing distribution across all four qualitative categories and Category A versus C/D for all CAD groups versus no CAD group are all *p *≥ 0.05.

Consistent with lab protocols, which include a longer warm up period on the treadmill, total test duration on the treadmill was longer but included a shorter period of time on the ramp than on the cycle ergometer (Table 2, *p *< 0.05). While peak $\dot{V}O_{2}$ occurred before peak work in 76% of tests, only 12% of all tests had a true plateau in p$\dot{V}O_{2}$, defined as greater than 30 s. Results in subsequent sections refer to assessment of the O_2_ pulse trajectory through p$\dot{V}O_{2}$ excluding any period of $\dot{V}O_{2}$ plateau unless otherwise noted.

### 3.3 O_2_ pulse trajectory assessment: Qualitative and quantitative metrics

Figure 2 presents the results of the qualitative assessment of the O_2_ pulse trajectories and the corresponding O_2_ pulse slopes. Most (64%) tests had normal linear augmentation throughout exercise (Category A). Of the remaining tests, 26% had O_2_ pulse trajectories that plateaued during late exercise (Category C) with slope decrement of 85 ± 36%, and 7% had O_2_ pulse trajectories that declined during late exercise (Category D) with slope decrement of 128 ± 19%. Few tests (3%) had O_2_ pulse that was flat throughout the ramp (Category B). Time on the ramp portion of the test was significantly shorter in these individuals versus patients with the other O_2_ pulse trajectories (308 ± 41 vs. 518 ± 170 s, *p *< 0.05), and all of these tests occurred in participants without CAD (Table 3). When O_2_ pulse trajectory was considered through peak work instead of p$\dot{V}O_{2}$, nine tests (9%) changed categories, with more having a late exercise decline (Category D, n = 12, 12%), and fewer having linear augmentation (Category A, n = 62, 60%).

### 3.4 O_2_ pulse trajectory assessment and coronary angiography results

Table 3 presents CPET parameters and coronary angiography results. Half of the participants underwent CCTA and half underwent ICA. Most participants (65%) had CAD, with OCAD in 23% and multi‐vessel OCAD in 9%. Ischemic changes on exercise ECG (Gibbons et al., 2002) were more common in participants with OCAD than those with no CAD (82% vs. 40%, *p *< 0.05). P$\dot{V}O_{2}$ and O_2_ pulse were similar among groups and remained supra‐normal even in those with multi‐vessel OCAD. The late exercise O_2_ pulse slope was similar in those with and without CAD, including OCAD. Flattening of the O_2_ pulse in late exercise relative to early exercise was numerically greater with increasing severity of CAD (Any CAD: −27 ± 85%, OCAD: −38 ± 57%, Multi‐vessel OCAD: −59 ± 44%), but was highly variable within and not significantly different between groups. Most participants with OCAD had normal linear augmentation in the O_2_ pulse (Category A, 67%). The proportion of participants with OCAD who had a plateau or decline in the O_2_ pulse (Category C or D, 33%) was similar to the group without any CAD (22%). Overall, there were no significant differences in the distribution of O_2_ pulse trajectories among the qualitative categories in those with any form of CAD versus those without CAD. Alternate categorization of O_2_ pulse curve shape using test data through peak work instead of p$\dot{V}O_{2}$ did not change this result.

### 3.5 Predictive performance of O_2_ pulse trajectory for CAD

In the evaluation for OCAD, the presence of ischemic ECG changes had an area under the receiver operating curve (AUC) of 0.69 and sensitivity (Se) of 82%, and specificity (Sp) of 57%. All O_2_ pulse parameters had poor predictive capability for OCAD. The AUC for a normal (Category A) versus abnormal (Category B, C, or D) O_2_ pulse trajectory was 0.52 (Se 33%, Sp 64%), and this was not improved by excluding tests with flat O_2_ pulse trajectory (Category A vs. Category C/D, AUC 0.51, Se 33%, Sp 67%) or classifying only those tests with O_2_ pulse decline as abnormal (Category A/B/C vs. D, AUC 0.52, Se 4%, Sp 93%). The AUC for late exercise O_2_ pulse slope was 0.55, with an optimal cut‐off of 0.48 ml/beat/min (Se 68%, Sp 41%). Similarly, the AUC for the proportional change in late exercise O_2_ pulse slope was 0.55, with an optimal cut‐off of 0.55 (Se 91%, Sp 18%). When O_2_ pulse parameters were added to the exercise ECG, the change in AUC was minimal (−0.01 to +0.02, all *p *≥ 0.05). When the O_2_ pulse trajectory was evaluated through peak work rather than p$\dot{V}O_{2}$, results were similar (range of AUC for O_2_ pulse parameters: 0.50–0.60, minimal (−0.01 to +0.03, *p *≥ 0.05) change when added to ECG).

### 3.6 Predictors of O_2_ pulse trajectory in the absence of obstructive CAD

Given the poor predictive ability of the O_2_ pulse parameters for OCAD, alternative predictors for a plateau or decline in the O_2_ pulse trajectory were explored in Table 4 among participants *without* OCAD. Participants with a plateau or fall in the O_2_ pulse reached a higher percent of predicted p$\dot{V}O_{2}$ (133 ± 31% vs. 114 ± 36% predicted, *p *< 0.05) and had a longer exercise ramp length (9.5 ± 3.2 vs. 8.0 ± 2.5 mins) as compared to those with normal linear O_2_ pulse augmentation. There were no other significant differences among O_2_ pulse trajectory groups in participants without OCAD.

**TABLE 4:** Characteristics of patients without obstructive CAD

Test characteristic	Qualitative assessment of O_2_P trajectory	
	Normal (Category A) (n = 51)	Plateau or decline (Category C or D) (n = 26)
Demographic characteristics		
Age (years)	53.5 (14.4)	58.2 (8.6)
Female	17 (33)	7 (27)
Baseline Anemia^b^, ^c^	4 (13)	1 (7)
Exercise ECG^d^		
Ischemic changes with exercise	18 (36)	14 (58)
CCTA or ICA Results		
Any CAD	26 (51)	18 (69)
Moderate CAD	6 (12)	4 (15)
Coronary Calcium Score (Agatston units)^c^	0 (0–20)	1 (0–168)
CPET testing characteristics		
Treadmill test	21 (41)	9 (35)
Ramp length to peak $\dot{V}O_{2}$ (min)	8.0 (2.5)	9.5 (3.2)^a^
Peak RER	1.17 (0.1)	1.19 (0.1)
Peak $\dot{V}O_{2}$ (ml/kg/min)	34.7 (13.9)	38.3 (9.6)
Peak $\dot{V}O_{2}$ percent predicted	114 (36)	133 (31)^a^
Peak work (Watts)^c^	207 (101)	264 (89)
$\dot{V}O_{2}$/Work (mL/min/W)^c^	8.6 (1.4)	9.5 (1.3)
Peak HR (bpm)	160 (29)	162 (19)
Peak HR, percent predicted	96 (13)	100 (10)
Peak O_2_ Pulse (ml/beat)	16.6 (4.9)	19.1 (6.7)
Peak exercise SBP	183 (35)	190 (32)
Peak exercise DBP	74 (13)	77 (12)
Mild EIAH (S_p_O_2_<95%)^c^	15 (31)	7 (32)
Moderate EIAH (S_p_O_2_<93%)^c^	3 (6)	1 (5)

Note

Data presented as n (%) or mean (SD).

Abbreviations: CAD, coronary artery disease; DBP, diastolic blood pressure; ECG, electrocardiogram; EIAH, exercise‐induced arterial hypoxia; HR, heart rate; RER, respiratory exchange ratio; SBP, systolic blood pressure.

^a^ *p*<0.05 for Category C/D vs. Category A Groups.

^b^ Defined as hemoglobin <13 g/dL in males or <12 g/dL in females.

^c^ Partial data for the following characteristics: Baseline Anemia (Category A: n = 31, Category C/D: n = 14), CAC scores (Category A: n = 31, Category C/D: n = 16), Peak Work and $\dot{V}O_{2}$/Work‐patients undergoing cycle tests (Category A: n = 30, Category C/D: n = 17), EIAH (Category A: n = 48, Category C/D: n = 22).

^d^ Percentage of those with ischemic ECG changes shown relative to those with interpretable ECGs (Category A: n = 50, Category C/D: n = 24).

## 4 DISCUSSION

This study sought to evaluate the diagnostic performance of quantitative and qualitative parameters defining O_2_ pulse trajectory for the diagnosis of OCAD in a physically fit population of patients, free of other relevant forms of cardiac disease, who were referred for clinical CPET. Key findings are summarized as follows. First, a plateau or decline in the O_2_ pulse trajectory during late exercise, as evaluated by several complementary qualitative and quantitative metrics, was not associated with the presence of OCAD on coronary angiography. Second, there was no significant improvement in diagnostic performance of CPET when O_2_ pulse metrics were added to exercise ECG. Third, among those without OCAD, an O_2_ pulse decline or plateau was associated with attainment of higher percentage of predicted p$\dot{V}O_{2}$ and longer exercise ramp time. Overall, these findings suggest that in a physically fit population, a plateau or decline in O_2_ pulse during late exercise is not a useful predictor of OCAD.

Current 2012 AHA/EACPR Clinical Recommendations for CPET specifically recommend the addition of O_2_ pulse trajectory assessment to standard exercise test variables in the diagnostic evaluation of patients with suspected myocardial ischemia (Committee & EACPR: Guazzi M, 2012). They cite in support of this recommendation the study by Belardinelli et al, which demonstrated that, in patients with known CAD and low average p$\dot{V}O_{2}$ (21 ml/kg/min), flattening of the O_2_ pulse and $\dot{V}O_{2}$ trajectories significantly improved the diagnostic performance of ECG‐only exercise testing for myocardial ischemia against a reference standard based on single photon emission computed tomography (SPECT) (Belardinelli et al., 2003). Subsequent investigations by this group and others have used an assortment of qualitative or quantitative assessments of the O_2_ pulse, have variably used SPECT or angiography to define OCAD, and have produced conflicting results (Belardinelli et al., 2003, 2014; De Lorenzo et al., 2017, 2018; Klainman et al., 1996; Laukkanen et al., 2006; Munhoz et al., 2007). Notably, published work that supports inclusion of O_2_ pulse trajectories in CPET guidelines has been limited to patients with low to normal physical fitness. Our goal was to overcome these limitations by evaluating the diagnostic performance of complementary qualitative and quantitative versions of this guideline‐recommended metric for OCAD, as diagnosed by angiography, in a physically fit cohort.

Our results may differ from that of others for several important reasons (Belardinelli et al., 2003, 2014; Munhoz et al., 2007). We found O_2_ pulse metrics lacked specificity for OCAD, which may be due to our narrower definition of OCAD as defined by angiography rather than SPECT and the higher fitness of our cohort as compared to other studies. A physiologic plateau in $\dot{V}O_{2}$ is more common in athletic individuals able to tolerate the discomfort required to reach a true maximal effort (Lucia et al., 2006). We attempted to reduce the impact of this issue in our physically fit cohort by evaluating O_2_ pulse metrics through peak $\dot{V}O_{2}$ rather than peak work. However, a plateau in O_2_ pulse may be, in part, a reflection of relative flattening in $\dot{V}O_{2}$ before it reaches peak. Alternately, others have suggested that an attenuated late exercise O_2_ pulse slope in athletes without OCAD may be explained by microvascular dysfunction (Van de sande et al., 2019). While we did observe a numerically higher proportion of ischemic ECGs in those athletes without OCAD who had an O_2_ pulse plateau or decline as compared to those with normal augmentation (Table 4), this result was not significant. We did not otherwise systematically assess for microvascular dysfunction in this study and this remains an important area of future scientific inquiry. Overall, a quarter of our cohort had a plateau or decline in O_2_ pulse but no OCAD, and the potential benign, physiologic nature of this finding in these individuals is supported by the fact that they were on average fitter than those with linear O_2_ pulse augmentation.

O_2_ pulse metrics in our study also lacked the sensitivity for OCAD that other studies have demonstrated (Belardinelli et al., 2003, 2014). In order for ischemic LV systolic dysfunction to be measurable by the O_2_ pulse, there must be enough myocardium subtended by obstructive disease to produce a meaningful change in exercise stroke volume. We defined our primary outcome as OCAD, but in those meeting criteria, the majority (63%) had single vessel disease. Given the small number of individuals with multi‐vessel disease in our study, we cannot exclude the possibility that O_2_ pulse metrics could have adequate sensitivity for disease affecting multiple coronary territories. Consistent with this finding, Munhoz et al. previously demonstrated that O_2_ pulse trajectory was impacted by extensive but not mild ischemia on SPECT (Munhoz et al., 2007). However, in the evaluation of a physically fit cohort, the identification of single‐vessel disease still has high clinical relevance given CAD is a leading cause of sudden cardiac death or arrest in masters endurance athletes (Kim et al., 2012).

Our finding that O_2_ pulse curve flattening or decline was relatively common in those without OCAD or any other identifiable cause for impaired SV challenges what constitutes normal O_2_ pulse augmentation in physically fit individuals. In patients without anemia or other medical conditions affecting O_2_ extraction, arteriovenous O_2_ difference augments linearly across the spectrum of exercise capacity (Stringer et al., 1985; Sullivan et al., 1989). However, whether linear augmentation in SV throughout graded exercise is the expected normal response is debated (Vella & Robergs, 2005). Some studies have suggested that normal individuals have robust augmentation in SV in early exercise followed by a relative plateau from mid to peak exercise (Astrand et al., 1964; Bevegard et al., 1963; Grimby et al., 1966; Higginbotham et al., 1986; Stringer et al., 1985, 2005; Trinity et al., 2012), but others have demonstrated that progressive linear increase in SV to peak exercise is possible in both trained (Gledhill et al., 1994; Zhou et al., 2001) and untrained individuals (Krip et al., 1997; Martino et al., 2002; Vella & Robergs, 2005). Overall, current evidence suggests that the normal SV response to graded exercise may vary on the basis of training, volume status, age, and sex (Vella & Robergs, 2005). Our results in physically fit individuals undergoing evaluation for OCAD and others’ results in healthy individuals suggest a mid to late exercise plateau in the O_2_ pulse may be on the spectrum of a normal exercise response.

This study has several limitations that merit discussion. First, our cohort was small and included tests performed on the treadmill and cycle ergometer. We combined the analysis because tests on cycle and treadmill shared similar late exercise O_2_ pulse characteristics and because this comprehensive assessment of the O_2_ pulse on both pieces of equipment may best reflect the clinical practice of many CPET labs. Second, the combination of both cycle ergometer and treadmill tests impacted the exact O_2_ pulse parameters that we chose to evaluate. For example, slopes were evaluated over time rather than work, which is not accurately definable across modalities, and we were not able to assess other trajectories, such as that of $\dot{V}O_{2}$/work or HR/work across the entire cohort. This choice was made in the context of our lab protocols that customize the ramp to target uniform test length, and we found that slopes defined by time were well reflected by our qualitative assessment of O_2_ pulse trajectory. Ultimately, we chose to evaluate a mix of quantitative and qualitative metrics to overcome this limitation, targeting those that reflect “real‐world” assessment of O_2_ pulse trajectory. Third, our cohort consisted of patients referred for clinical evaluation who underwent CPET for a variety of clinical indications and was further selected by those who underwent angiography. Furthermore, some patients were anemic or had mild to moderate EIAH. Therefore, the sensitivity and specificity of O_2_ pulse metrics may be different in an unselected population of healthy, physically fit individuals, or patients undergoing CPET specifically for evaluation of CAD. Finally, we evaluated for CAD using a combination of CCTA and ICA, relied on clinical reporting, and noted a low proportion (33%) of invasive angiograms utilized physiologic testing with FFR or iFR, all raising the possibility of misclassification of CAD severity. However, OCAD was defined by ICA in the vast majority of cases (83%), and we noted that moderate disease was not more common in those with an O_2_ pulse decline or plateau (Table 4), lessening concern that systematic underestimation of CAD severity impacted results.

In conclusion, we found that the application of O_2_ pulse metrics to identify a late exercise plateau, as it is recommended by guidelines, was limited by low sensitivity and specificity for OCAD in our physically fit cohort and did not improve the diagnostic performance of ECG. Though O_2_ pulse metrics may still have a role in the evaluation for ischemia‐induced LV dysfunction in those with average or lower exercise capacity, our results suggest that the use of O2 pulse trajectories in isolation or in combination with the ECG may have limited diagnostic utility in the prediction of OCAD in physically active clinical populations who undergo CPET. Though CPET remains an enormously useful clinical test for the evaluation of cardiopulmonary disease (Balady et al., 2010), diagnosis and management of suspected OCAD may benefit from integration of CPET results with other clinical factors and cardiac testing.

## CONFLICT OF INTEREST

None.
